# Socioeconomic status (SES) and 30-day hospital readmissions for chronic obstructive pulmonary (COPD) disease: A population-based cohort study

**DOI:** 10.1371/journal.pone.0216741

**Published:** 2019-05-21

**Authors:** Andrea S. Gershon, Deva Thiruchelvam, Shawn Aaron, Matthew Stanbrook, Nicholas Vozoris, Wan C. Tan, Eunice Cho, Teresa To

**Affiliations:** 1 Department of Medicine, Sunnybrook Health Sciences Centre, Toronto, Ontario, Canada; 2 Institute for Clinical Evaluative Sciences, Toronto, Ontario, Canada; 3 Clinical Epidemiology Program, Ottawa Hospital Research Institute, Ottawa, Ontario, Canada; 4 Asthma & Airway Centre, Toronto Western Hospital, Toronto, Ontario, Canada; 5 Department of Medicine, St. Michael’s Hospital, Toronto, Ontario, Canada; 6 Centre for Heart Lung Innovation, University of British Columbia, Vancouver, British Columbia, Canada; 7 Department of Medicine, University of Toronto, Toronto, Ontario, Canada; 8 Child Health Evaluative Sciences, Hospital for Sick Children, Toronto, Ontario, Canada; Cardiff University, UNITED KINGDOM

## Abstract

**Background:**

Patients with chronic obstructive pulmonary disease (COPD) are more likely to be readmitted than patients with other chronic medical conditions, yet knowledge regarding such readmissions is limited. We aimed to determine factors associated with readmission within 30 days of a COPD hospitalization or death with an emphasis on examining aspects of socioeconomic status and specific comorbidities.

**Methods:**

A population-based cohort study was conducted using health administrative data from Ontario, Canada. All hospitalizations for COPD between 2004 and 2014 were considered. The primary exposures were socioeconomic status as measured by residential instability (an ecologic variable), and comorbidities such as cardiovascular disease and cancer. Other domains of socioeconomic status were considered as secondary exposures. Logistic regression with generalized estimating equations was used to examine the effect of exposures, adjusting for other patient factors, on 30-day readmission or death.

**Results:**

There were 126,013 patients contributing to 252,756 index COPD hospitalizations from 168 Ontario hospitals. Of these hospitalizations, 19.4% resulted in a readmission and 2.8% resulted in death within 30 days. After adjusting for other factors, readmissions or death were modestly more likely among people with the highest residential instability compared to the lowest (OR 1.05, 95% CI 1.01–1.09). Comorbidities such as cardiovascular disease and cancer, as well as other aspects of low socioeconomic status also increased readmission or death risk.

**Interpretation:**

Socioeconomic status, measured in various ways, and many comorbidities predict 30-day readmission or death in patients hospitalized for COPD. Strategies that address these factors may help reduce readmissions and death.

## Introduction

Chronic obstructive pulmonary disease (COPD) affects approximately 10% of adults worldwide [1], is the third leading cause of death [2] and accounts for significant health services use [3, 4]. About one in five patients discharged from hospital for COPD gets readmitted within 30 days [5–8] with a rate higher than other chronic medical conditions [5]. In an effort to optimize quality of care, many countries have developed programs aimed directly at preventing such unplanned readmissions [9–12]; however, interventions to reduce COPD-specific readmissions have been met with limited success [13, 14]. A possible reason is because strategies designed to reduce readmission have placed little emphasis on upstream factors, specifically low socioeconomic status (SES) and comorbidities.

We are aware of only three large population studies that examined the impact of low socioeconomic status on COPD hospital readmissions [6, 15, 16]; however, none examined varying aspects of socioeconomic status. Likewise, few studies have considered comorbidity [16–18], and those that have, missed many that would be relevant. More knowledge of both these factors would inform strategies to reduce COPD readmission. Finally, studies of COPD readmissions in general have been limited by studying readmissions after asthma as well as COPD hospitalizations [6, 16, 17]; by only focusing on hospitalizations with COPD as a primary diagnosis instead of all hospitalizations that people with COPD made related to their disease—such as for pneumonia [19, 20]; and by not considering readmissions to hospitals other than the one of the original admission [19]. In addition, there is little knowledge of factors affecting readmission in countries with health care insurance that covers most basic medical needs of the population and where direct costs are not likely to be a barrier, like Canada.

Therefore to learn more about how SES and comorbidities impact 30 day readmission after a COPD hospitalization in a large, complete COPD population in Canada—a country with health care insurance for the population that covers most medical needs, we conducted the current study.

## Materials and methods

### Study design, setting and data sources

We conducted a population study using health administrative data from all individuals in Ontario, Canada between 2004 and 2014 to characterize all-cause readmissions within 30 days of a COPD hospitalization discharge, particularly the impact of SES and different types of comorbidity. Ontario is the largest province of Canada with a diverse population of about 14 million. Most healthcare expenses are paid by the government of Ontario.

Three health administrative databases were used for this study that included information on patient demographics, all health services provided by physicians and all acute care hospitalizations in Ontario (for more details, see **Supplemental Text**). The research ethics committee at Sunnybrook Health Sciences Centre approved this study. An approval number is not available as providing approval numbers is not part of the standard processes. A waiver of informed consent was obtained as the data was analyzed anonymously.

#### Study population

All individuals aged 35 or greater in Ontario who had one or more unplanned, acute care hospitalization where COPD contributed significantly to length of stay between January 1, 2004 and November 30, 2014 were included. Validation studies have shown that identification of people with COPD in the databases used can be done with good sensitivity and specificity. [21, 22] Hospitalizations for people with both COPD and asthma were among those included, however, hospitalizations where asthma (and not COPD) contributed significantly to length of stay were not included, as they have been in some previous studies, because of our focus was on COPD. Hospitalizations where patients were ineligible for Ontario healthcare insurance; where there were missing values on key measures such as patient identifier, discharge date or discharge disposition; where length of stay was an outlier; where patients were transferred to a second acute care hospital on discharge; or where patients died were excluded. Hospitalizations that occurred within 30 days of a previous hospitalization were considered readmissions and not index hospitalizations. For more details see **Supplemental materials**.

#### Exposure, outcome and co-variables

SES was characterized using the four domains of the validated Ontario Marginalization Index that capture processes by which individuals and groups are prevented from fully participating in society [23]. **Residential instability** considers the proportion of people living in an area who are alone or youth, as well as number of persons per dwelling, number of people living in apartments, and number of people who are married. Other measures of SES considered were **Material deprivation**, **Ethnic concentration**, and **Dependency** (for details, see **Supplemental Text**). These variables were determined ecologically using patients’ residential postal code and information from 2006 Canadian Census data, and expressed in quintiles. The components of this index have not been validated in people with COPD.

Because there is high correlation between the four SES domains, we designated residential instability as our primary exposure and then substituted in each of the other domains in secondary analyses. We also considered people’s neighborhood income quintile.

Comorbidity was determined from the index hospitalization record and characterized using components of the Charlson index that describe individual comorbidities [24, 25].

Since death is a clinically significant, unfavourable competing risk for hospital readmissions, the primary outcome was an unplanned hospital readmission for any cause within 30 days of the index hospitalization or death. For more details on readmission, see **Supplemental material**.

A number of covariates were also considered including patient demographics, COPD-related factors, prior healthcare use, and index-hospitalization-related factors.

### Analyses

Readmission rate was calculated as the proportion of patients who were readmitted to any hospital within 30 days. We examined why people returned by noting how many readmissions were due to COPD versus other causes. The latter were further categorized based on ICD-10 codes [26]. We examined when patients returned to hospital by noting daily distribution of readmissions from the day of initial discharge.

To examine risk factors for readmission or death, we considered all index hospitalizations for each patient and their 30-day readmissions or death. Multivariable logistic regression with generalized estimating equations was used to identify risk factors associated with 30-day readmissions or death adjusting for all covariates. We considered but rejected using a competing risk analysis as this would be dependent on using survival analysis, which we did feel applied because when during the 30 days after discharge a death or readmission occurred is not nearly as clinically relevant as if it occurred. As some patients had more than one index hospitalization and were often clustered within hospitals, both patient and hospital correlations were accounted for. Five separate multivariable models were run, one for each SES domain. Missing data were handled using a complete case approach. All analyses were performed using SAS version 9.3 (SAS Institute, Cary, North Carolina, USA).

Additional analyses including an examination of COPD specific readmissions were performed (for details, see **Supplemental Text**).

## Results

### Study cohort

**Fig 1:**
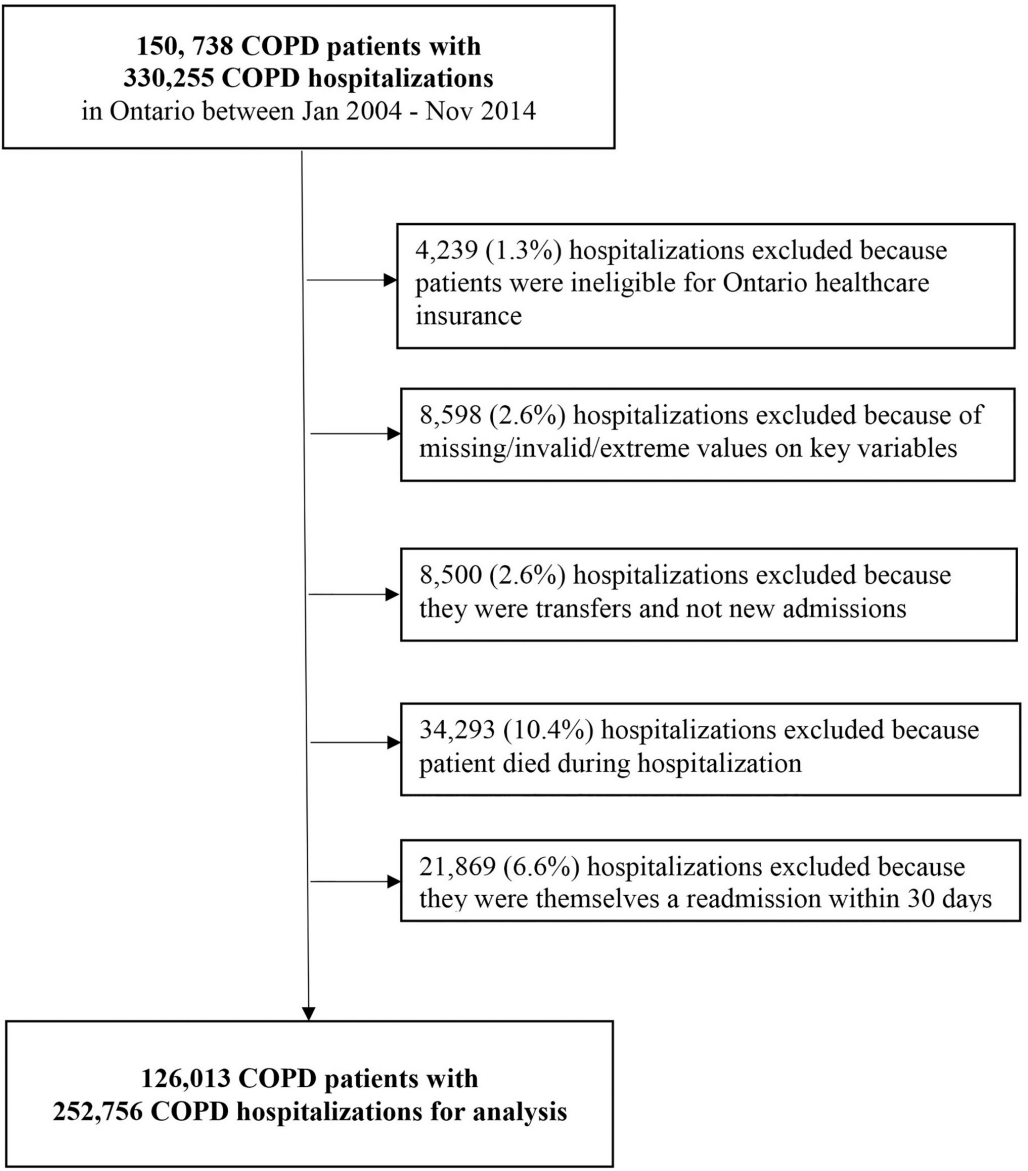
Flow chart of index hospitalizations.

After exclusions, 126,013 COPD patients discharged from 252,756 COPD hospitalizations from 168 Ontario hospitals between January 2004 and November 2014 were available for analysis (Fig 1). Most patients were 65 and older at time of index discharge and most were males. More than half had one (58.5%), 19.8% had two and 21.7% had three or more qualifying index hospitalizations during the study period. Baseline characteristics of index hospitalizations are presented in Table 1. Lower SES as reflected by greater marginalization was associated with older age, female sex, urban residence, longer duration of COPD, a prior hospitalization within 6 months, and a greater number of prior emergency department (ED) visits. Readmission rates by various comorbidities are presented in **e-Table 1**.

**Table 1:** Characteristics of patients with COPD hospitalizations by socioeconomic status as reflected by residential instability, a domain of the Ontario Marginalization Index.

	Residential Instability					
Characteristic	Quintile 1 (least marginalized)	Quintile 2	Quintile 3	Quintile 4	Quintile 5 (most marginalized)	*p*-value^a^
**Number of index hospitalizations**	22,233	36,292	47,840	59,902	86,490	
**Readmission rate (%)**	18.6	18.6	18.7	19.5	20.3	< .001
**Demographic, %**						
Age, years						
35–44	0.3	0.7	0.5	0.6	0.6	< .001
45–54	3.2	3.7	4.2	4.8	5.3	
55–64	13.4	13.7	13.7	15.1	15.3	
65–74	28.5	28.9	28.2	27.1	27.2	
75–84	38.6	37.4	36.9	35.5	34.3	
85+	15.9	15.7	16.5	16.9	17.2	
Male	56.6	53.6	52.8	50.4	46.7	< .001
Urban residence (versus rural)	81.8	74.2	70.4	81.0	94.6	< .001
**COPD-related, %**						
Duration of COPD diagnosis						
Less than 1 year	7.2	6.7	6.4	6.3	6.0	< .001
1–5 years	19.4	18.9	18.4	18.1	18.0	
More than 5 years	73.4	74.4	75.2	75.6	76.0	
COPD Specialist care^b^	71.9	65.9	65.2	65.2	70.0	< .001
**Comorbidity, %**						
Acute myocardial infarction	5.2	5.2	5.1	5.6	6.1	< .001
Congestive heart failure	21.4	20.4	20.2	20.8	20.7	0.003
Peripheral vascular disease	2.5	2.3	2.3	2.1	2.5	< .001
Cerebrovascular disease	2.1	1.8	1.8	2.0	2.1	< .001
Dementia	4.2	3.9	4.1	4.4	4.5	< .001
Connective tissue / rheumatic disease	1.0	1.0	0.9	0.9	0.8	0.003
Peptic ulcer disease	0.4	0.4	0.4	0.4	0.5	0.023
Mild liver disease	0.7	0.6	0.7	0.8	1.0	< .001
Moderate or severe liver disease	0.2	0.2	0.2	0.2	0.2	0.914
Diabetes without complications	10.4	10.1	10.0	10.6	10.3	0.024
Diabetes with complications	13.8	12.8	12.7	13.0	13.3	< .001
Hemiplegia or Paraplegia	0.4	0.4	0.4	0.5	0.5	0.013
Renal disease	6.6	5.8	5.8	5.8	5.6	< .001
Primary cancer	4.9	5.3	5.1	4.9	4.6	< .001
Metastatic cancer	1.8	1.8	1.7	1.7	1.6	0.083
Active asthma	24.7	22.3	21.7	22.3	23.6	< .001
Respiratory failure	7.8	6.9	6.3	6.7	7.8	< .001
**Prior healthcare use, %**						
Prior hospitalization						
Less than or equal to 6 months prior	26.2	25.8	26.0	27.2	27.9	< .001
6 months to 5 years prior	36.4	36.4	37.0	36.9	37.3	
More than 5 years or none	37.4	37.8	37.0	35.9	34.8	
Number of prior ED visits in previous year						
0	42.8	40.7	40.0	39.0	39.0	< .001
1	25.5	25.6	25.5	25.1	25.1	
2	14.2	14.3	14.2	14.5	14.5	
3	7.6	8.1	8.2	8.3	8.2	
4 or more	10.0	11.3	12.1	13.1	13.2	
Intensive care unit stay in previous year	2.3	2.1	2.1	2.2	2.1	0.281
**Index hospitalization-related, %**						
Admitted through the ED	94.2	93.3	93.5	94.1	95.2	< .001
Length of index hospitalization, days						
1	1.1	1.0	1.0	1.1	1.4	< .001
2	4.9	4.8	4.9	5.0	5.2	
3	8.9	9.1	9.1	8.9	8.7	
4 to 6	31.3	30.9	31.2	30.8	30.1	
7 to 13	34.9	35.3	34.6	35.1	34.3	
14 or more	18.9	18.9	19.2	19.1	20.4	
Discharge disposition						
Transfer to long-term care / other institution	13.2	12.7	14.4	15.4	17.3	< .001
Home with support services	24.7	25.4	26.4	27.3	27.9	
Home	61.4	61.0	58.4	56.3	53.4	
Left against medical advice	0.7	0.8	0.8	1.0	1.4	

ED = Emergency Department

^a^Overall P-value testing for differences in a characteristic across residential instability. Univariate comparisons were done using one way ANOVA for continuous variables, Kruskal-Wallis test for continuous skewed variables, chi-squared tests for categorical variables and Cochran-Armitage trend test for ordinal variables.

^b^COPD specialist referred to respirology, internal medicine, or geriatric medicine specialists.

**Fig 2:**
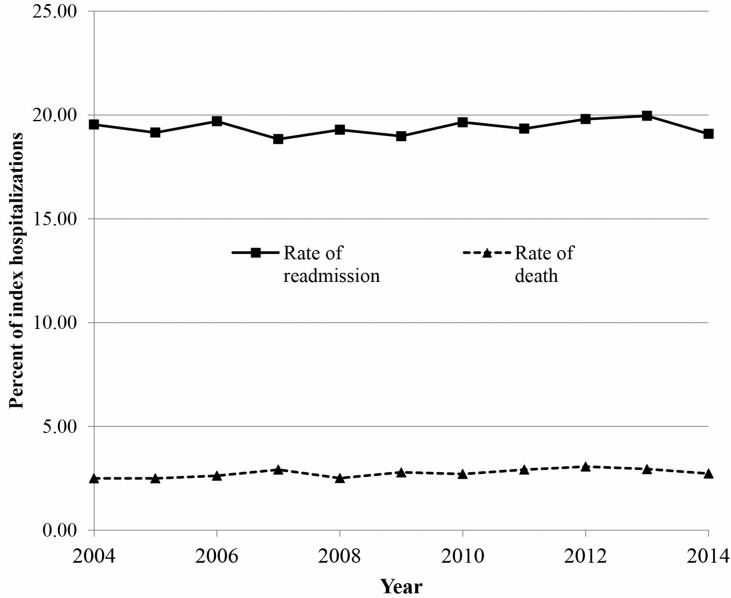
Rates of all-cause readmission and death within 30 days of a COPD hospital discharge.

**Fig 3:**
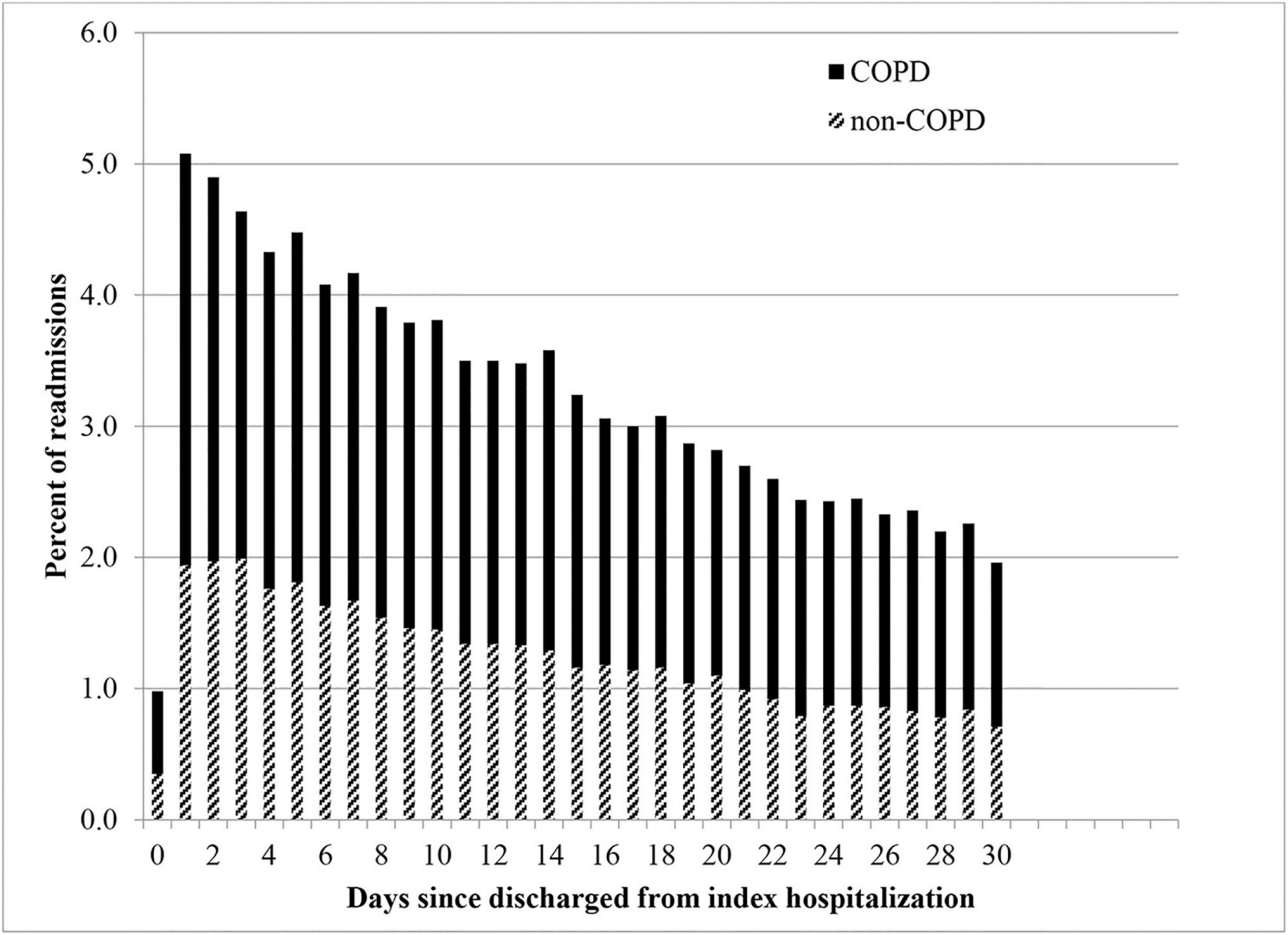
Time to readmission for patients readmitted after a hospitalization for COPD by cause of readmission.

Of the 252,756 index COPD hospitalizations, 49,046 (19.4%) resulted in a readmission and 6,961 (2.8%) resulted in death within 30 days. Crude rates of readmission and death remained relatively stable over the study period (Fig 2). Among the 49,046 readmissions, 3,847 (7.8%) led to a second readmission and 163 (4.2%) lead to a third within 30 days of the previous. About 62% of readmissions were for COPD, the remaining were for other causes (Table 2), most notably cardiovascular disease. The median time to readmission was 12 days (interquartile range (IQR): 6–20 days). The highest percent of readmissions occurred the day after discharge with amounts gradually decreased thereafter (Fig 3). Consistently, about two thirds of the readmissions each day were for COPD.

**Table 2:** Causes of 30-day readmission among patients with a COPD hospitalization.

Cause of readmission	Percent
COPD	61.9
Non-COPD	38.1
Diseases of the circulatory system	9.8
Diseases of the respiratory system other than COPD	4.5
Diseases of the digestive system	4.0
Infectious and parasitic diseases	2.6
Injury, poisoning and other external causes	2.3
Diseases of the genitourinary system	1.8
Neoplasms	1.8
Endocrine, nutritional and metabolic diseases	1.6
Mental and behavioral disorders	0.9
Other	8.8

#### Risk factors for all-cause readmissions and death

**Fig 4:**
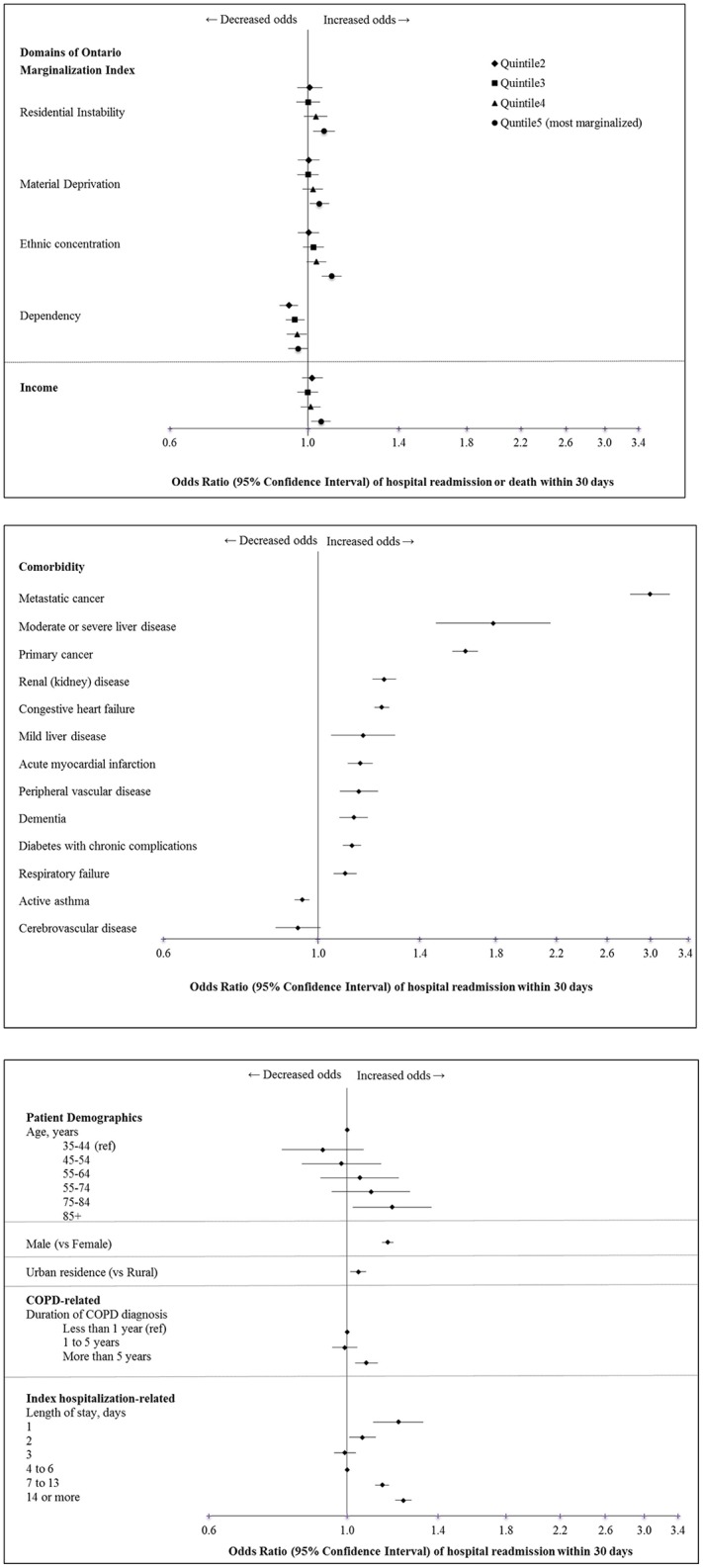
Adjusted odds ratios of A. socioeconomic factors, B. comorbidities and C. patient factors associated with 30-day readmission or death after a COPD hospitalization.

Residential instability and all other domains of SES marginalization were found to be risk factors for all-cause 30-day hospital readmission or death in unadjusted and adjusted analyses (**e-Table 2**; Table 3; Fig 4). Specifically, patients living in areas of highest residential instability (OR 1.05, 95% CI 1.01–1.09), highest material deprivation (OR 1.04, 95% CI 1.00–1.07), highest ethnic concentration (OR 1.06, 95% CI 1.02–1.10) had more readmissions and death than their least marginalized counterparts. In contrast, patients living in areas of lowest dependency had higher readmission rates than patients living in areas with slightly higher dependency (OR 0.95, 95% CI 0.91–0.99), but this association was attenuated with increasing levels of dependency.

**Table 3:** Adjusted odds ratios (with 95% CI and p-values) of factors associated with different 30-day outcomes for Individuals with chronic obstructive pulmonary disease^a^.

Characteristic	All-cause readmission or death				All-cause readmission				COPD-specific readmission			
	Odds Ratio	Lower 95% confidence interval	Upper 95% confidence interval	*p*-value	Odds Ratio	Lower 95% confidence interval	Upper 95% confidence interval	*p*-value	Odds Ratio	Lower 95% confidence interval	Upper 95% confidence interval	*p*-value
**Residential Instability**												
Lowest	1.00				1.00				1.00			
Next least marginalized	1.02	0.97	1.06	0.435	1.01	0.96	1.06	0.751	1.02	0.96	1.08	0.469
Middle	1.00	0.96	1.04	0.970	1.00	0.96	1.05	0.975	1.04	0.98	1.10	0.171
Next most marginalized	1.02	0.98	1.06	0.399	1.03	0.99	1.07	0.185	**1.06**	**1.00**	**1.12**	**0.038**
Most marginalized	**1.05**	**1.01**	**1.09**	**0.025**	**1.06**	**1.02**	**1.11**	**0.004**	**1.09**	**1.04**	**1.15**	**<0.001**
**Demographic**												
Age, years												
35–44	1.00				1.00				1.00			
45–54	0.91	0.79	1.06	0.243	0.92	0.79	1.07	0.253	0.91	0.75	1.10	0.336
55–64	0.98	0.85	1.13	0.788	0.99	0.85	1.14	0.857	1.00	0.83	1.19	0.960
65–74	1.05	0.91	1.21	0.524	1.05	0.90	1.21	0.556	1.06	0.88	1.27	0.548
75–84	1.09	0.95	1.26	0.228	1.08	0.93	1.25	0.330	1.03	0.86	1.24	0.743
85+	**1.18**	**1.02**	**1.37**	**0.025**	1.10	0.95	1.28	0.203	0.99	0.82	1.18	0.873
Male	**1.16**	**1.14**	**1.19**	**<0.001**	**1.15**	**1.12**	**1.17**	**<0.001**	**1.17**	**1.14**	**1.21**	**<0.001**
Urban	**1.04**	**1.01**	**1.07**	**0.004**	**1.05**	**1.02**	**1.08**	**0.0019**	**1.08**	**1.04**	**1.12**	**<0.001**
**COPD-related**												
Duration of COPD												
Less than 1 year	1.00				1.00				1.00			
1–5 years	0.99	0.95	1.04	0.724	1.00	0.95	1.05	0.93	**1.18**	**1.10**	**1.26**	**<0.001**
More than 5 years	**1.07**	**1.03**	**1.12**	**<0.001**	**1.09**	**1.04**	**1.14**	**<0.001**	**1.45**	**1.36**	**1.54**	**<0.001**
COPD Specialist care	**1.13**	**1.10**	**1.15**	**<0.001**	**1.15**	**1.12**	**1.18**	**<0.001**	**1.14**	**1.10**	**1.17**	**<0.001**
**Co-morbidity**												
Myocardial Infarction	**1.15**	**1.10**	**1.20**	**<0.001**	**1.14**	**1.09**	**1.19**	**<0.001**	1.00	0.94	1.06	0.989
Congestive Heart Failure	**1.24**	**1.21**	**1.27**	**<0.001**	**1.22**	**1.18**	**1.25**	**<0.001**	**1.08**	**1.05**	**1.12**	**<0.001**
Peripheral Vascular Disease	**1.14**	**1.08**	**1.22**	**<0.001**	**1.14**	**1.07**	**1.22**	**<0.001**	1.06	0.97	1.16	0.168
Cerebrovascular Disease	0.94	0.87	1.01	0.079	0.95	0.87	1.02	0.172	**0.83**	**0.75**	**0.93**	**0.0008**
Dementia	**1.13**	**1.07**	**1.18**	**<0.001**	1.05	1.00	1.11	0.062	0.98	0.92	1.05	0.605
Connective tissue / rheumatic disease	0.97	0.88	1.08	0.585	0.99	0.89	1.10	0.802	0.92	0.80	1.06	0.231
Ulcers of the Digestive System	0.98	0.85	1.14	0.799	1.05	0.90	1.23	0.517	**0.73**	**0.57**	**0.92**	**0.007**
Mild Liver Disease	**1.16**	**1.05**	**1.29**	**0.006**	**1.18**	**1.06**	**1.31**	**0.003**	0.99	0.86	1.14	0.897
Diabetes without complications	1.00	0.96	1.03	0.812	1.01	0.97	1.05	0.630	0.94	0.90	0.98	0.006
Diabetes with complications	**1.12**	**1.09**	**1.15**	**<0.001**	**1.15**	**1.12**	**1.19**	**<0.001**	1.01	0.97	1.05	0.593
Hemiplegia or Paraplegia	1.13	0.97	1.31	0.111	1.11	0.93	1.31	0.243	1.01	0.80	1.27	0.960
Renal Disease	**1.25**	**1.20**	**1.30**	**<0.001**	**1.24**	**1.19**	**1.29**	**<0.001**	1.05	0.99	1.11	0.113
Primary Cancer	**1.63**	**1.56**	**1.70**	**<0.001**	**1.43**	**1.37**	**1.50**	**<0.001**	**1.10**	**1.04**	**1.17**	**0.002**
Moderate or Severe Liver Disease	**1.78**	**1.48**	**2.16**	**<0.001**	**1.62**	**1.32**	**1.99**	**<0.001**	0.78	0.56	1.10	0.155
Metastatic Cancer	**3.00**	**2.81**	**3.20**	**<0.001**	**1.99**	**1.84**	**2.14**	**<0.001**	**1.19**	**1.07**	**1.33**	**0.002**
Active asthma	**0.95**	**0.93**	**0.97**	**<0.001**	**0.97**	**0.95**	**1.00**	**0.046**	**1.07**	**1.04**	**1.10**	**<0.001**
Respiratory failure on index admission	**1.09**	**1.05**	**1.14**	**<0.001**	**1.05**	**1.01**	**1.10**	**0.012**	**1.18**	**1.13**	**1.24**	**<0.001**
**Prior healthcare use**												
Prior hospitalization												
Less than or equal to 6 months prior	**1.56**	**1.52**	**1.60**	**<0.001**	**1.58**	**1.54**	**1.63**	**<0.001**	**2.39**	**2.30**	**2.49**	**<0.001**
6 months to 5 years prior	**1.28**	**1.24**	**1.31**	**<0.001**	**1.31**	**1.27**	**1.34**	**<0.001**	**1.66**	**1.61**	**1.72**	**<0.001**
More than 5 years or none	1.00				1.00				1.00			
Number of prior ED visits												
0	1.00				1.00				1.00			
1	**1.18**	**1.15**	**1.22**	**<0.001**	**1.20**	**1.17**	**1.24**	**<0.001**	**1.11**	**1.07**	**1.15**	**<0.001**
2	**1.42**	**1.38**	**1.47**	**<0.001**	**1.46**	**1.41**	**1.51**	**<0.001**	**1.33**	**1.28**	**1.39**	**<0.001**
3	**1.60**	**1.54**	**1.66**	**<0.001**	**1.65**	**1.59**	**1.72**	**<0.001**	**1.48**	**1.40**	**1.55**	**<0.001**
4 or more	**2.22**	**2.14**	**2.29**	**<0.001**	**2.31**	**2.23**	**2.39**	**<0.001**	**2.02**	**1.93**	**2.11**	**<0.001**
Prior intensive care unit stay	**1.10**	**1.03**	**1.17**	**0.008**	**1.11**	**1.03**	**1.19**	**0.004**	**1.12**	**1.03**	**1.22**	**0.009**
**Index hospitalization-related**												
Admitted to index hospital through ED	**1.26**	**1.21**	**1.32**	**<0.001**	1.21	1.16	1.27	**<0.001**	1.47	1.37	1.57	**<0.001**
Length of index hospitalization, days												
1	**1.21**	**1.10**	**1.33**	**<0.001**	**1.15**	**1.04**	**1.26**	**0.006**	**1.20**	**1.07**	**1.35**	**0.002**
2	**1.06**	**1.01**	**1.11**	**0.023**	1.04	0.99	1.09	0.132	**1.10**	**1.04**	**1.17**	**0.002**
3	0.99	0.95	1.03	0.696	0.98	0.95	1.03	0.437	**1.01**	**0.96**	**1.06**	**0.769**
4 to 6	1.00				1.00				1.00			
7 to 13	**1.14**	**1.11**	**1.17**	**<0.001**	**1.13**	**1.10**	**1.16**	**<0.001**	**1.08**	**1.05**	**1.12**	**<0.001**
14 or more	**1.23**	**1.20**	**1.27**	**<0.001**	**1.18**	**1.14**	**1.21**	**<0.001**	1.03	0.99	1.07	0.171
Discharge disposition												
Transfer to long term care/Other	**1.42**	**1.38**	**1.47**	**<0.001**	**0.93**	**0.90**	**0.97**	**<0.001**	**0.89**	**0.85**	**0.93**	**<0.001**
Home with support services	**1.33**	**1.29**	**1.36**	**<0.001**	**1.30**	**1.27**	**1.34**	**<0.001**	**1.28**	**1.24**	**1.32**	**<0.001**
Home	1.00				1.00				1.00			
Left against medical advice	**2.02**	**1.85**	**2.20**	**<0.001**	**2.01**	**1.84**	**2.19**	**<0.001**	**2.11**	**1.90**	**2.34**	**<0.001**

^a^Analyses adjusted for adjusted for age, gender, home location, duration of COPD, COPD specialist care, comorbidities, previous hospitalizations, previous ED visits, previous intensive care unit admission, admission to hospital through ED, length of index hospitalization, hospital discharge disposition, and year of index hospitalization.

Of all comorbidities studied, cardiovascular disease (myocardial infarction, congestive heart failure, peripheral vascular disease), liver disease, dementia, diabetes with chronic complications, renal disease, and cancer were associated with increased risk of readmission in unadjusted and adjusted analyses (**e-Table 2**; Fig 4). Of note, active asthma, and cerebrovascular disease were not found to be associated with increased risk in adjusted analysis.

In addition to SES factors and comorbidities, being older, being male, living in an urban area, longer duration of COPD, and short or longer length of index hospitalization were also significant predictors of all-cause readmissions (Fig 4). Other significant risk factors included leaving the index hospital against medical advice, being discharged home with support services, being admitted to the hospital through the ED, having a recent hospitalization or more prior ED visits, a previous intensive care unit stay, and being cared for by a COPD specialist.

Additional analyses are reported in the **Supplemental Text**.

## Discussion

We conducted a population-based cohort study to characterize the impact of SES and comorbidity on 30-day COPD readmissions and found that in a jurisdiction with health care insurance that cover most basic medical needs of the population and where direct costs are not likely to be a barrier, about one in five COPD index hospital discharges resulted in an unplanned readmission or death, that about 60% of readmissions were due to COPD, and that several SES domains and certain comorbidities were associated with an increased risk for readmission or death. We also confirmed that being male, living in an urban area, and previous hospitalizations were risk factors for readmission or death. These results can be used to inform risk prediction tools to identify people with COPD who are at high risk of readmission and target strategies to reduce their risk[27].

We found that patients who were highly marginalized were more likely to be readmitted to hospital or die which is consistent with other COPD studies in different populations using different measures of SES [6, 15, 28]. Our relative increases in risk associated with SES indices were modest; however, since COPD is one of the most common causes of hospital admission, they equate to thousands of affected patients. It extends previous findings by confirming SES as a risk factor in a large, complete COPD population covered by provincial healthcare insurance. Proposed reasons for this are numerous, including that marginalized individuals are less able to take time off work to attend medical visits and are more likely to have a lower levels of health literacy. These factors should be considered when creating strategies to address readmission. They should also be considered when implementing policies, such as the Hospital Readmissions Reduction Program in the U.S., that reduce funding to hospitals with high readmission rates—for fear of penalizing hospitals that serve disadvantaged populations [9, 10].

Dependency was the one domain of marginalization that did not follow the trends of the other SES domains. Although there was a trend toward more readmissions in areas of greatest dependency overall, this trend was reversed in people living in the lowest and second lowest dependency areas. We hypothesize that this is because dependency is partially driven by labor force participation, or conversely, unemployed people living in the home. Some unemployment could mean people available to be caregivers of people with COPD and keep them out of hospital, whereas at a lot of unemployment could drive substantial financial insecurity that overwhelms the benefit of a stay-at-home caregiver. This is an area deserving of further study.

Knowing residential instability affects COPD readmissions is important to understanding the experiences of our patients but it is not a risk factor that is easily modified. Nonetheless, being aware of it on hospital discharge, asking patients about their housing situation and informing them of resources that might be available to help them could benefit them and, eventually, reduce hospital readmissions.

Our study extended the findings of previous studies that showed comorbidity to be a risk factor for readmission [6, 16, 18, 29], by demonstrating that many different conditions, including some not found before, were risk factors for readmission, and some appeared to be COPD-specific. For example, patients with comorbid mild liver disease were 16% more likely to get readmitted for any cause or die, although not specifically for COPD. Addressing these high-risk conditions during discharge planning could help prevent readmissions. The risk factors we found are consistent with those specified in the validated PEARL (Previous admissions, extended MRC dyspnoea score, Age, Right-sided heart failure and Left-sided heart failure) prognostic score for COPD readmissions and death even though the study that established that score included several clinical measures that we did not have access to. The additional risk factors found in our study may have been due to its larger sample size and ability to detect smaller increases in risk [20].

Our finding that one in five COPD hospital discharges resulted in a readmission is consistent with previous population-based studies [5–7]. We also found that—despite advances in healthcare—the rate of readmission to be stable over the last decade. This trend is consistent with the one found by Shah et al. in the U.S. Medicaid/Medicare population [6]. Finally, we found that many factors found to be associated with readmission in previous studies were also relevant to patients in a health care system where all patients were insured for most medical needs.

About two-thirds of index COPD hospitalization readmissions in our study were due to COPD, with the highest rate of return occurring on the day after discharge. Shah et al. reported only half of readmissions to be due to COPD [6]. However, they also included index hospitalizations for asthma—which are less likely to be associated with readmission for COPD.

Our study also extended the findings of previous readmission studies by reporting and considering rate of death (a competing risk) during the follow-up period [6, 17, 30]. As with 30-day readmission rate, 30-day mortality rate is also a measure of hospital care reflecting, in part, effective care and discharge planning. Since death and readmission following hospitalization share many risk factors, preventing hospital readmission may also help reduce death.

The strengths of our study were its ability to study an entire, large COPD population over a 10 year period with complete information on all hospitalizations. Our study also has limitations that need to be mentioned. First, we identified people with COPD physician claims, however physicians are known to underdiagnose COPD [31] thus we might have missed some people with COPD who would have contributed to the readmission rate. Misclassification could have also caused us to identify people with COPD and other comorbidities who did not have those conditions. Second, our measures of SES, the four domains of the Ontario Marginalization Index, were determined ecologically and not at the individual level. It is possible that individual level measures—such as those obtained by a clinician when assessing a patient—may have a stronger influence on readmission rates. However, previous work has shown that ecological SES correlates well with individual SES and can be used in population-based cohort studies [32, 33]. Finally, our results might not be generalizable to healthcare settings without universal health care insurance. However, our rates and risk factors for readmission were similar to those in jurisdictions without universal coverage, such as the United States, suggesting our findings would also reflect other circumstances in these areas.

In conclusion, our population study of COPD readmissions revealed about one in five index hospitalizations resulted in an unplanned readmission, that about 60% of readmissions were due to COPD, and that several SES domains and certain comorbidities were risk factors for readmission or death. These results can be used to develop risk assessment tools to identify high risk patients prior to discharge and provide them with discharge planning that focuses on factors, such as housing conditions and specific comorbidities, that might not otherwise be addressed.

## Supporting information

S1 File Supplemental material- characterizing 30-day hospital readmissions. (PDF)

## Abbreviations

COPD: chronic obstructive pulmonary disease
SES: socioeconomic statu**s**
