# Supplementary material for: Socioeconomic status (SES) and 30-day hospital readmissions for chronic obstructive pulmonary (COPD) disease: A population-based cohort study
Source: PLoS One. 2019 May 21;14(5):e0216741. doi: 10.1371/journal.pone.0216741 (PMC6528994; doi:10.1371/journal.pone.0216741)
Supplement: S1 File — (PDF) [file pone.0216741.s001.pdf]

Socioeconomic status (SES) and 30-day hospital readmissions for chronic obstructive pulmonary (COPD) disease: a population-based cohort study

Supplementary File

Supplemental Text

Methods

Study Data Sources

The Registered Persons Database contains basic demographic information including sex and date of birth. The Ontario Health Insurance Plan Physician Claims database contains information on all services provided by fee-for-service physicians and "shadow-billings" for physicians paid under alternate payment plans. The Canadian Institute of Health Information Discharge Abstract database contains information on all acute care hospitalizations. These datasets were deterministically linked using unique encoded identifiers, with 100% success for the patients studied, and analyzed at the Institute for Clinical Evaluative Sciences. The study was approved by the institutional review board at Sunnybrook Health Sciences Centre, Toronto, Ontario, Canada. The full dataset creation plan is available from the authors upon request.

Study Population

All individuals aged 35 or greater in Ontario who had one or more unplanned, acute care hospitalizations where COPD contributed significantly to length of stay between January 1, 2004 and November 30, 2014 were included. Unplanned hospitalizations were those where patients' initial status was recorded as urgent or emergent. COPD hospitalizations were those with International Classification of Disease 10 codes J41-J44 as most responsible diagnosis or as an admitting diagnosis or pre-admit comorbidity that contributed at least 24 hours to a patient's length of stay Hospitalizations where patients were ineligible for Ontario healthcare insurance; where there were missing values on key measures such as patient identifier, discharge date or

discharge disposition; where length of stay was an outlier (greater than the 99th percentile); where patients were transferred to a second acute care hospital on discharge; or where patients died were excluded. Individuals could have more than one index hospitalization. Hospitalizations that occurred within 30 days of a previous hospitalization were considered readmissions and not index hospitalizations. The exception to this was in the calculation of repeated readmissions.

Readmissions

Readmissions with pregnancy, childbirth, or puerperium as the most responsible diagnosis were not considered. If there were multiple readmissions within the 30-day period, only the first was used to calculate and predict readmission rates. All, however, were counted to examine rates of multiple readmissions within 30 days. COPD-specific readmissions were considered in secondary analyses

Exposure Outcomes

SES was characterized using the four domains of the validated Ontario Marginalization Index that capture processes by which individuals and groups are prevented from fully participating in society.[20] **Residential instability** considers the proportion of people living in an area who are alone or youth, as well as number of persons per dwelling, number of people living in apartments, and number of people who are married. **Material deprivation**, another domain, considers the proportion of people with no high school graduation, who are from lone parent families, who are unemployed, and/or who are low income. **Ethnic concentration** considers the proportion of people who are recent immigrants and visible minorities. Finally, **Dependency** considers the ratio of seniors and children to working age adults and labor force participation.

Additional Analyses

To determine if results were consistent when the competing risk of death was not considered, we repeated the main analysis with the outcome of 30-day all-cause readmissions alone. To determine if COPD specific readmission differed from readmissions due to any cause, we performed additional analyses substituting the former for the later. Secondary analyses were completed examining readmission rates or death within 90 days and stratifying the primary analysis by younger (under 65 years) and older age.

Results

Additional Analyses

Risk factors for all-cause readmissions (but not death) and their magnitudes (including the four domains of SES marginalization and income quintile) were similar to the risk factors for all-cause readmissions and death except dementia was no longer a significant risk factor (**Table 3**). Risk factors for COPD-specific readmissions and their magnitudes (including the four domains of SES marginalization and income quintile) were largely similar to all-cause readmissions and death, except for comorbidities where only congestive heart failure, asthma and cancer were significantly associated with increased COPD readmissions. People age 65 and older seemed less affected by SES factors than those under 65 (**e-Table 3**). Some comorbidities but not socioeconomic factors appeared to be risk factors for 90 day readmissions or death. (**e-Table 4**).

Supplemental Tables

e-Table 1: Readmission rate or death among people with and without various comorbidities.

	Readmission rate or death among people (%)		
Comorbidity	With comorbidity	Without comorbidity	p-value
Cerebrovascular Disease	1,167 (23.6%)	54,840 (22.1%)	0.015
Congestive Heart Failure	14,443 (27.7%)	41,564 (20.7%)	<.001
Connective tissue / rheumatic disease	490 (21.5%)	55,517 (22.2%)	0.468
Dementia	2,878 (26.6%)	53,129 (22.0%)	<.001
Diabetes without complications	5,504 (21.1%)	50,503 (22.3%)	<.001
Diabetes with complications	8,855 (26.8%)	47,152 (21.5%)	<.001
Hemiplegia or Paraplegia	298 (26.5%)	55,709 (22.1%)	<.001
Mild Liver Disease	529 (25.7%)	55,478 (22.1%)	<.001
Moderate or Severe Liver Disease	185 (36.3%)	55,822 (22.1%)	<.001
Acute Myocardial Infarction	3,778 (26.6%)	52,229 (21.9%)	<.001
Peptic Ulcer Disease	263 (23.0%)	55,744 (22.2%)	0.498
Peripheral Vascular Disease	1,532 (25.9%)	54,475 (22.1%)	<.001
Primary Cancer	3,770 (30.5%)	52,237 (21.7%)	<.001
Metastatic Cancer	1,888 (44.0%)	54,119 (21.8%)	<.001
Renal Disease	4,317 (29.3%)	51,690 (21.7%)	<.001
Active Asthma	12,853 (22.3%)	43,154 (22.1%)	0.511
Respiratory failure	4,487 (24.9%)	51,520 (21.9%)	<.001

e-Table 2. Non-adjusted odds ratios (with 95% CI and p-values) of residential stability and various comorbidities and 30-day readmission or death individuals with chronic obstructive pulmonary disease

	All-cause readmission or death			
**Characteristics**	**OR**	**Lower 95% confidence interval**	**Upper 95% confidence interval**	***p*-value**
**Residential Instability**				
Lowest	1.00			
Next least marginalized	1.01	0.97	1.04	0.7448
Middle	1.00	0.97	1.03	0.9888
Next most marginalized	1.03	1.00	1.07	0.0482
Most marginalized	1.07	1.04	1.10	<0.0001
**Co-morbidities**				
Myocardial Infarction	1.22	1.18	1.26	<0.0001
Congestive Heart Failure	1.34	1.32	1.37	<0.0001
Peripheral Vascular Disease	1.18	1.12	1.23	<0.0001
Cerebrovascular Disease	1.08	1.02	1.14	0.0045
Dementia	1.24	1.20	1.28	<0.0001
Connective tissue / rheumatic disease	0.97	0.90	1.06	0.4968
Ulcers of the Digestive System	1.05	0.94	1.17	0.4222
Mild Liver Disease	1.14	1.06	1.23	0.0008
Diabetes without complications	0.94	0.92	0.97	<0.0001
Diabetes with complications	1.26	1.23	1.29	<0.0001
Hemiplegia or Paraplegia	1.21	1.10	1.34	0.0002
Renal Disease	1.36	1.33	1.40	<0.0001
Primary Cancer	1.44	1.40	1.48	<0.0001
Moderate or Severe Liver Disease	1.65	1.46	1.85	<0.0001
Metastatic Cancer	2.10	2.02	2.17	<0.0001
Active asthma	0.97	0.95	0.98	0.0003
Respiratory failure	1.13	1.10	1.16	<0.0001

e-Table 3. Adjusted odds ratios (with 95% confidence intervals and p-values) of association between residential instability and various comorbidities and 30-day Readmission or death for individuals with chronic obstructive pulmonary disease, by age less than or over 65 years

	**Residential instability**							
	**Age less than 65 years**				**Age more than or equal to 65 years**			
**Characteristic**	**Odds Ratio**	**Lower 95% confidence interval**	**Upper 95% confidence interval**	***p*-value**	**Odds Ratio**	**Lower 95% confidence interval**	**Upper 95% confidence interval**	***p*-value**
**Residential Instability**								
Lowest	1.00				1.00			
Next least marginalized	1.01	0.90	1.14	0.828	1.02	0.97	1.07	0.485
Middle	1.00	0.89	1.11	0.952	1.00	0.95	1.04	0.909
Next most marginalized	1.05	0.94	1.16	0.407	1.01	0.97	1.05	0.691
Most marginalized	1.09	0.99	1.21	0.084	1.03	0.99	1.08	0.137
**Co-morbidity**								
Myocardial Infarction	1.19	1.06	1.34	0.005	1.14	1.09	1.19	<0.001
Congestive Heart Failure	1.14	1.06	1.24	0.001	1.26	1.23	1.29	<0.001
Peripheral Vascular Disease	1.05	0.86	1.27	0.633	1.15	1.08	1.23	<0.001
Cerebrovascular Disease	0.90	0.71	1.14	0.372	0.94	0.87	1.02	0.131
Dementia	1.10	0.76	1.60	0.617	1.13	1.08	1.19	<0.001
Connective tissue / rheumatic disease	1.02	0.79	1.31	0.906	0.96	0.85	1.07	0.462
Ulcers of the Digestive System	1.05	0.69	1.58	0.833	0.97	0.83	1.14	0.708
Mild Liver Disease	1.23	1.05	1.44	0.009	1.07	0.92	1.23	0.401
Diabetes without complications	1.01	0.94	1.09	0.705	0.98	0.95	1.02	0.377
Diabetes with complications	1.18	1.08	1.28	<0.001	1.10	1.07	1.14	<0.001
Hemiplegia or Paraplegia	1.36	0.97	1.92	0.078	1.05	0.89	1.24	0.541
Renal Disease	1.42	1.23	1.63	<0.001	1.24	1.19	1.29	<0.001
Primary Cancer	1.95	1.74	2.19	<0.001	1.57	1.50	1.65	<0.001
Moderate or Severe Liver Disease	1.84	1.39	2.44	<0.001	1.75	1.35	2.27	<0.001
Metastatic Cancer	3.43	2.92	4.02	<0.001	2.89	2.69	3.11	<0.001
Active asthma	0.98	0.93	1.03	0.389	0.94	0.91	0.96	<0.001
Respiratory failure	1.04	0.96	1.12	0.377	1.10	1.06	1.15	<0.001

e-Table 4. Adjusted odds ratios (with 95% confidence internal and p-values) of residential instability and various comorbidities and all-cause readmission or death within 90 days for individuals with chronic obstructive pulmonary disease

	All-cause readmission or death within 90 days			
**Characteristic**	**Odds Ratio**	**Lower 95% confidence interval**	**Upper 95% confidence interval**	***p*-value**
**Residential Instability**				
Lowest (reference)	1.00			
Next least marginalized	1.01	0.96	1.06	0.677
Middle	0.99	0.94	1.03	0.538
Next most marginalized	0.99	0.95	1.04	0.769
Most marginalized	1.02	0.98	1.07	0.269
**Co-morbidity**				
Myocardial Infarction	1.15	1.10	1.20	<0.001
Congestive Heart Failure	1.26	1.23	1.29	<0.001
Peripheral Vascular Disease	1.15	1.07	1.23	<0.001
Cerebrovascular Disease	0.94	0.87	1.02	0.137
Dementia	1.11	1.05	1.16	<0.001
Connective tissue / rheumatic disease	0.97	0.87	1.09	0.604
Ulcers of the Digestive System	0.99	0.85	1.16	0.886
Mild Liver Disease	1.18	1.05	1.32	0.006
Diabetes without complications	1.00	0.96	1.03	0.873
Diabetes with complications	1.11	1.08	1.15	<0.001
Hemiplegia or Paraplegia	1.11	0.95	1.30	0.185
Renal Disease	1.27	1.21	1.32	<0.001
Primary Cancer	1.69	1.61	1.76	<0.001
Moderate or Severe Liver Disease	1.90	1.56	2.31	<0.001
Metastatic Cancer	3.13	2.92	3.35	<0.001
Active asthma	0.93	0.91	0.96	<0.001
Respiratory failure	1.09	1.04	1.13	<0.001

Explanation as to why this study does not have a Research Ethics Board Number

ICES is a prescribed entity under section 45 of *Ontario's Personal Health Information Protection Act* (PHIPA). Section 45 is the provision that enables analysis and compilation of statistical information related to the management, evaluation and monitoring of, allocation of resources to, and planning for the health system. Section 45 authorizes health information custodians to disclose personal health information to a prescribed entity, like ICES, without consent for such purposes.

Section 45 contrasts with that of section 44 of PHIPA, under which projects require review by a Research Ethics Board. Although projects conducted wholly under section 45, by definition, do not require REB review, the REB at Sunnybrook Health Sciences Centre – ICES' REB of Record – has agreed to help ICES achieve a higher ethical standard for the use of administrative and other data held at ICES. This is done using a retrospective review process whereby, at the end of each calendar year, ICES submits a report on the projects undertaken that year to the REB for review and approval. The REB may select individual projects from the report for review of the corresponding proposals and privacy impact assessments developed and archived at ICES. The above project was included in the 2012 annual report to the REB.

Because of the way the retrospective review process takes place, *individual projects are not given approval numbers.*

In addition to informing Sunnybrook's REB of projects undertaken at ICES, the REB is aware that the Information and Privacy Commissioner of Ontario reviews ICES' policies, practices and procedures for privacy protection and data security tri-annually. These include policies, practices and procedures that require review and approval of every project by ICES' Privacy Office. ICES was approved by the Commissioner for a fourth time in 2014.
